# Approaches to reduce false positives and false negatives in the analysis of microarray data: applications in type 1 diabetes research

**DOI:** 10.1186/1471-2164-9-S2-S12

**Published:** 2008-09-16

**Authors:** Jian Wu, Nataliya I Lenchik, Ivan C Gerling

**Affiliations:** 1 Department of Neurology, Xuan Wu Hospital, Capital Medical University, Beijing,China; 2 Department of Medicine, University of Tennessee Health Science Center, Memphis, TN, 38104, USA; 3 Research Service, Veterans Affairs Medical Center, Memphis, TN, USA; 4 Division of Endocrinology, University of Tennessee Health Science Center, VAMC Research 151, 1030 Jefferson Avenue, Memphis, TN 38104, USA

## Abstract

**Background:**

As studies of molecular biology system attempt to achieve a comprehensive understanding of a particular system, Type 1 errors may be a significant problem. However, few investigators are inclined to accept the increase in Type 2 errors (false positives) that may result when less stringent statistical cut-off values are used. To address this dilemma, we developed an analysis strategy that used a stringent statistical analysis to create a list of differentially expressed genes that served as "bait" to "fish out" other genes with similar patterns of expression.

**Results:**

Comparing two strains of mice (NOD and C57Bl/6), we identified 93 genes with statistically significant differences in their patterns of expression. Hierarchical clustering identified an additional 39 genes with similar patterns of expression differences between the two strains. Pathway analysis was then employed: 1) identify the central genes and define biological processes that may be regulated by the genes identified, and 2) identify genes on the lists that could not be connected to each other in pathways (potential false positives). For networks created by both gene lists, the most connected (central) genes were interferon gamma (IFN-γ) and tumor necrosis factor alpha (TNF-α). These two cytokines are relevant to the biological differences between the two strains of mice. Furthermore, the network created by the list of 39 genes also suggested other biological differences between the strains.

**Conclusion:**

Taken together, these data demonstrate how stringent statistical analysis, combined with hierarchical clustering and pathway analysis may offer deeper insight into the biological processes reflected from a set of expression array data. This approach allows us to 'recapture" false negative genes that otherwise would have been missed by the statistical analysis.

## Background

The comprehensive analysis of gene microarray datasets, each containing thousands of genes, face statistical "Catch 22" between Type 1 (false negatives) and Type 2 (false positive) errors. In traditional "linear paradigm" research, Type 2 errors can be devastating as they provide false conclusions upon which further work and conclusions may be based. Therefore, investigators often adopt rigorous statistical criteria to reduce Type 2 errors and like to see experiments repeated (preferably by other investigators) before believing a conclusion.

Conclusions based on analysis of microarray often serve only as the initial foundation for subsequent confirmation and more traditional avenues of research. As a result, investigators using expression arrays tend to be less concerned about the potential for a small number of false positives. However, a high number of false negatives may result from the failure to recognize the involvement of specific biological processes or molecular networks in the physiological system under study. As a result investigators using comprehensive expression arrays often find themselves in this "Catch 22" situation. If they use traditional statistical methods, with conservative multiple test corrections, there is potential for a large number of Type 1 errors. If they use "generous" statistical criteria to reduce Type 1 errors, they generate a list of genes so large that it cannot be comprehensively analyzed in a meaningful way.

Presently, we investigated the use of a novel data mining strategy. First, a microarray dataset was subjected to analysis using very stringent statistical criteria to define spleen leukocyte genes whose expression was different between the two strains of mice. Second, this set of "core" differentially expressed genes, was then used as "bait" in a cluster analysis to pull out another set of "peripheral" genes whose expression patterns was very similar to that of the core genes, although these "peripheral" genes had not been identified by the initial statistical criteria. This was done by subjecting the whole set of expression array data to hierarchical clustering and then examining the resulting "gene tree" for sub-branches that contained a high percentage of core genes.

Finally, we conducted network analyses on both lists of genes as a way to sort out genes that are not biologically connected to the other genes on the list and to define the molecular networks that were represented within each list of genes. This allowed us to compare the physiological processes represented by the two resulting networks.

## Results

We analyzed expression array data from 20 samples of spleen leukocytes with 10 samples from the NOD and 10 samples from C57BL/6 female mice. Half of the samples from each strain were collected at two weeks of age and half at four weeks. First, we conducted a two-way ANOVA on the 26,530 probesets to determine genes with statistically significant (p < 0.001) differences between strains and age groups. The stringent Bonferroni multiple correction test was used to reduce the number of false positives. With the Bonferroni correction, we performed one test for each probeset and then ranked the 26,530 p-values. For 99.9% confidence we divide 0.001 by 26530 to get 3.77 × 10^-8^. If any p = value is lower than 3.77 × 10^-8 ^it passes the Bonferroni multiple correction test. Thus, use of Bonferroni for mitigating false error rates in data sets with large numbers of tests resulted in highly conservative p-values and a very small number of false positives. This analysis found 93 probesets with statistically significant differences between strains. Because genes expressed differently at the two ages, but not between the two strains, likely represent normal developmental process, we focused on the 93 probesets that varied between the strains. The 93 probesets can be divided into two groups. One group is the 14 probesets with relatively high expression in NOD and low expression in C57BL/6 mice. The second group contains the 79 probesets with relatively low expression in NOD and high expression in C57BL/6 mice.

### Hierarchical clustering

**Figure 1:**
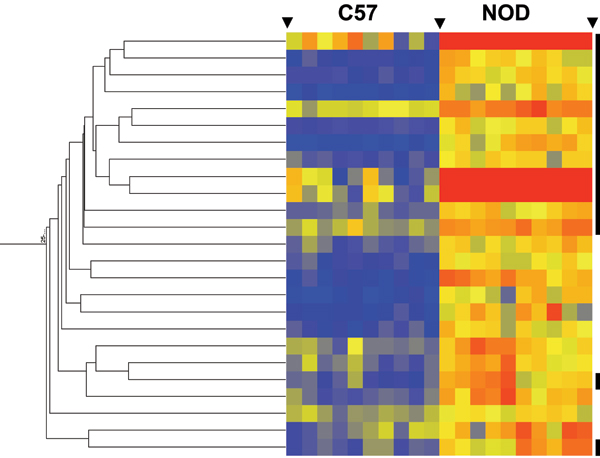
Hierarchical cluster of 25 probesets with relatively high expression in NOD and low expression in C57BL/6 mice. The 14 probesets with significant differences between the strains (P < 0.001) are marked by black bars on the right side of the figure. The intensity of the node color indicates the degree of increase (red) or decrease (blue) of the gene expression signal relative to the mean signal intensity.

Although our use of the Bonferroni correction reduces the number of false positives on our gene lists, it increases the occurrence of Type 1 errors (false negatives). To capture genes whose expression followed the same pattern seen for the lists of 14 and 79 probesets described above, we conducted hierarchical clustering on 26530 probesets and organized them into a "gene-tree". Using the 14 probesets with relatively high expression in NOD to guide us towards sub-branches within the gene-tree, we found an extra 11 probesets that had a similar expression pattern. The combined total of 25 probesets (14 plus 11) were themselves organized using the Gene Tree feature in GeneSpring program (Figure 1). The 14 probesets with significant differences between strains differences (P < 0.001), are marked by black bars. Statistical analysis of the 11 genes (without multiple test correction) indicated strain differences with a p-value of 0.00022 or less. This result demonstrates that all 25 probesets clearly have higher levels of expression in NOD than C57BL/6 mice. By the same approach, we found an extra 28 probesets with the same expression pattern as the 79 probesets with lower expression in NOD than C57BL/6 mice (data not shown). In total, the hierarchical cluster analysis yielded an additional 39 probesets that show expression differences between the two strains.

### Molecular networks

The list of 93 probesets was uploaded to the Ingenuity server which contains information about millions of known connections between individual genes. Our list of 93 probesets represented 46 different focus genes listed in the Ingenuity Pathway Knowledge Base (IPKB) The remaining probesets represented genes about which the IPKB did not contain information and could not connect to other genes.

**Figure 2:**
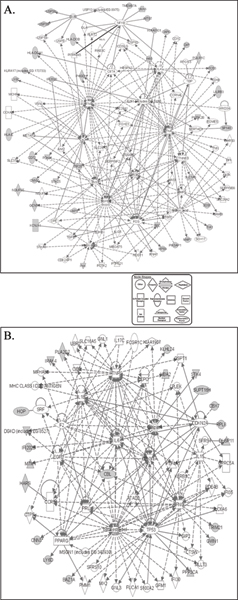
Network analysis by Ingenuity Pathway Analysis. A: Merged network created from the 93 probesets that have highly significant strain differences by 2-way ANOVA. The network contains 38 focus genes (i.e., genes originating from the list of 93 probesets) indicated by gray icons. B: Merged network created by the 39 additional probesets from the hierarchical analysis.

The database created three major networks. We merged those 3 networks and created a single network (Figure 2A). In Figure 2A, there are 38 focus genes (derived from the 93 probesets) and 67 non-focus genes indicated by gray and white icons, respectively. The number of connections of the nine most connected genes (central genes) is presented in Table 1 IFN-γ and TNFα had the greatest number of connections (35 and 32, respectively).

**Table 1:** Central genes in networks from figure 2 and their number of connection within the network.

93 Probesets Merged Network		39 Probesets Merged Network	
Gene	# of Connection	Gene	# of Connection

IFNG	35	TNF	26
TNF	32	IFNG	24
TGFB1	30	TP53	23
FOS	19	IL1B	21
IL4	18	IL6	16
CTNNB1	13	CDKN1A	14
NFYB	12	IL15	13
E2F1	12	F2	13
BMP2	8	EGF	13

The list of 39 additional probesets obtained by hierarchical clustering analysis also was subjected to Ingenuity Pathways Analysis (IPA). The 39 probesets represented 24 genes found in IPKB. IPA created two major networks.

The merged network from those two is presented in Figure 2B, and it contain 21 focus genes and 51 non-focus genes. Table 1 also shows the number of connections of the nine most connected genes (central genes) in the network from Figure 2B. The largest numbers of connections again involved IFN-γ (24) and TNF-α (26).

## Discussion

In the NOD animal model of autoimmune (Type 1) diabetes, the first sign of pathology is seen at about 5-weeks of age [1,2]. As the animals get older, the infiltration of the pancreas with leukocytes, and the subsequent destruction of insulin producing β-cells, gets progressively worse until the mice become diabetic. To gain further insight into mechanisms and pathways involved, our study focused on the molecular abnormalities at 2–4 weeks of age [3,4]. We used comprehensive molecular characterization (molecular phenotyping) at both the transcriptome and proteome levels [3,5,6].

In order to gain insight from such large datasets, it is important to develop an effective strategy for data mining and analysis. The problems associated with analyzing expression data from a very large number of genes (discussed previously) have lead to development of alternative statistically approaches and tools. Cluster analysis and principal component analysis have become popular tools for the analysis of expression array data [7-12]. When combined with further downstream data processing and/or data mining, these can be effective tools to gain more insight from a large dataset. However, many investigators are uncomfortable with the use of these clustering methods to define sets of genes that are differentially expressed when comparing different groups of biological samples. They want to know that the difference is statistically significant, i.e. has not just simply appeared by chance. To take advantage of the strengths and compensate for the weaknesses of each of these data analysis approaches, we have combined them. First, we used a traditional statistical analysis (ANOVA) with very stringent criteria and a multiple test correction to define a set of 93 genes that showed highly significant differences in expression levels between the two strains of mice. We also conducted a hierarchical clustering analysis of the gene expression dataset. Then, we examined the resulting gene tree to find genes with expression patterns very similar to (i.e., clustering together with) the 93 genes. This process defined another 39 genes where the differences between the expression levels of the two strains did not reach the stringent criteria of statistical significance.

We have found that the best way to gain insights from such an analysis is to evaluate the biological "relatedness" of the genes by conducting a pathway analysis. The Ingenuity data-mining tool uses knowledge from the literature to evaluate how best to connect all the genes on a list (focus genes) to each other with the help of some non-focus genes. This approach does have a bias toward genes for which there is a lot of published data, and is not likely to connect a gene for which little more than its name is known. However, for the purpose of gaining a broad understanding of the underlying biological phenomena these newly defined genes cannot be used. They must be mined individually by different, and much more time-consuming, approaches. Another advantage of using the pathway analysis to mine large gene lists is that it may help sort out false positives. Any false positive gene on a list is not likely to be connected to the other genes because it would not likely be part of the underlying biological processes. Therefore, that gene is also less likely to become a part of the network created by the Ingenuity server.

When we conducted pathway analysis on the list of 93 probesets that showed highly significant differences between the two strains of mice, we created a network in which IFN-γ and TNF-α were the most central (i.e., had the most connections to other genes in the network). The third highly connected gene in that network, TGF-β1, is a multifunctional growth factor involved in many different immunological processes [13,14]. Both IFN-γ and TNF-α are important for the process of reducing autoimmunity in the developing immune system [15-17]. Furthermore, when combined with IL1-β these cytokines are known to be directly cytotoxic to insulin producing beta-cells. This has been hypothesized to be an important mechanism of autoimmune diabetes [18-20].

When we evaluated the network created by the 39 genes identified only by cluster analysis, we again found that TNF-α and INF-γ have the most connections to other genes in the network. This confirmed that genes on the two lists were highly connected to each other not just by expression patterns, but also by the underlying physiology. Yet some of the other central genes in the network created from the list of 39 genes were different and may help to further define and interpret the network created from the list of 93 genes. First, we should consider IL1-β, which, as mentioned above, is cytoxic to β-cells. Second, one of the many roles of TGF-β1 is that it is an anti-inflammatory cytokine [21]. The network from the list of 39 genes contains three pro-inflammatory cytokines (IL1β, IL6, and IL15) among its most central genes. This would suggest that not only regulation of development of autoimmune T-lymphocytes, but also regulation of inflammatory responses may define the critical differences in the immune system of young mice that will, or will not, develop autoimmune diabetes at a later age. This suggests that a more detailed study of the interaction of regulatory processes for inflammation and autoreactive T-lymphocytes is merited.

## Conclusion

This paper demonstrates how stringent statistical analysis can be combined with hierarchical clustering to gain much better insight from a set of expression array data. The combination identifies critical false negative genes missed by the statistical analysis, such that conclusions and models based on statistical data can be refined and expanded.

## Methods

### Collection of tissues and gene expression analysis

The specific methods for the collection of spleen leukocytes and analysis of gene expression on Affymetrix expression arrays have been described elsewhere [4]. This paper also describe the process of filtering through the 44,000 probesets present in the MOE 403A and MOE 403B expression arrays to identify the 26,530 probesets that are expressed at least once in the spleen leukocyte samples. This list of 26,530 probesets was subjected to statistical and cluster analysis.

### Statistical and cluster analysis

The expression data produced by the MAS 5.0 software was imported into GeneSpring software (version 7.3.1, Silicon Genetics, Redwood, CA) and analyzed. The dataset consists of a total of 20 samples that can be divided into four groups if both strain (NOD and C57BL/6) and age (2 week and 4 week) are considered. We conducted a two-way ANOVA test (P < 0.001) with Bonferroni multiple test corrections to produce lists of differentially expressed genes with significant differences between strains and age groups. We also conducted hierarchical clustering with Pearson's Correlation Coefficient on all 26,530 probesets. The list of 26,530 probesets was organized into a "gene-tree" where genes were grouped according to similarities in their expression patterns across all 20 samples. We used the genes from the two-way ANOVA to guide us towards sub-branches that contained clusters of genes with either high or low expression in NOD mice. We collected all genes from each sub-branch to create a new list of genes that followed the same expression pattern as the genes with statistically significant differences in expression between the strains.

### Analysis of molecular networks using Ingenuity

The gene lists were analyzed through the use of Pathways Analysis (Ingenuity Systems, ) as previously described [4]. The list of genes containing gene identifiers (i.e. Affymetrix ID of probesets) was uploaded into the application. Each gene identifier was mapped to its corresponding gene object in the Ingenuity Pathways Knowledge Base. Networks of these genes were then algorithmically generated based on their connectivity. We used IPA to visualize and explore molecular networks created from the lists of probesets. The networks created from a list consist of genes from that list (called focus genes) and genes that are not on the list but help connect all the genes together in a network (called non-focus genes). In Figure 2, the focus and non-focus genes are depicted with gray and white icons, respectively. Due to the server's restriction that no network can contain more than 35 genes, both gene lists were used to create multiple networks. This division into multiple networks is usually just an artifact, and the database often contains information demonstrating that there are multiple connections between genes from different networks. The IPA allows the easy merging of individual networks. We used the "merge networks" feature to create a single network. We then counted the connections for the central genes which had connections to at least eight genes in the merged network.

## Competing interests

The authors declare that they have no competing interests.

## Authors' contributions

ICG designed the research and data-analysis approaches. JW and NIL collected samples and gene expression data. JW conducted all data analysis and data mining. JW and ICG wrote the paper.
